# Associative Effort

**Published:** 1871-03

**Authors:** 


### ASSOCIATIVE EFFORT.

What a sense of weariness and dissatisfaction takes possession of the mind, after wading through the proceedings of the dental society, or after sitting out the meeting. And yet, there is no good reason for that result, for, without pausing here to either discuss or even state the causes, it is one that must inevitably flow from the present prevailing condition of dental associations.

In these days when the revelations of the microscope are common enough to no longer excite the wonder of scientific men, it seems that any pretense towards knowledge, would be made in the high light of reality and truth only, and that fundamental principles alone would engage the attention of earnest and studious men; but that the very contrary more nearly approximates to the general status is a fact that the intelligent will at once recognize and regret.

Take for instance, the discussions in any dental association, and what are they? Helps even to a clearer understanding of fundamental truth? Hardly, but sharp-pointed personalities; eulogies of instruments and materials; unkind criticisms of somebody's methods, and a vigorous ventilation of egotism—all served up in a gravy of badly compounded English and uncooked grammer. Occasionally a ray of light is thrown upon some subject, dear to the best interests of the profession, but it is soon obscured in gaseous exhaltations, and remembered only as a shining point in the dusky firmament.

The association can confer benefit upon the members only in proportion as it rises above the little personal things, the mechanical details, and the petty interests of practice, and in order to reach out and aspire it is absolutely necessary that every member fob his favorite and egotistical "I think," and come down with his honest knowledge, coined out of faithful study and careful observation.

The important interests of education and legislation are too much neglected. The very existence of the profession calls for more scrupulosity of the one, and a larger application of the other. As long as mercenary amateurs and peripatetic scamps can roam the country, and while imposing upon the intelligence of the people, practice the barbarous and obsolete method of extraction as a cure for toothache, it is time associative effort was made to keep the mercenary out, or being in, elevate them to a better and in the end a more valuable knowledge. With the unconscionable scamp it may be more difficult to deal, but it has been demonstrated in Ohio, that such legislation can be had, as will protect the people against his dangerous and destructive practices, in spite of his plausibility and their ignorance.

What is greatly needed, we apprehend, is a more sympathetic and united effort in dental associations. Anything calculated to lower the dignity of the body, such as personalities that too often cheapen the proceedings, is vitiating, and will destroy all usefulness. It is with the judgment and experience of the occupant of the floor that the body has to do, and not with his tool chest and the rules of his practice; but if these are intruded upon the body, it should be the duty of the chairman to remind the speaker that the dental profession has a code of ethics.

An association of scientific men, is like a powerful galvanic battery, which sends the concentrated force through one medium for the accomplishment of a definite purpose, but if the cups in the battery shall insist upon having the medium split up into so many wires to carry their divided force, how little will be effected!

An idea once advanced should receive a careful and good natured consideration, and in order to secure that, no hap-hazard thought should be advanced. That which shall benefit the profession at large, and invest it with nobler purpose and purer character, should engage the attention of associations everywhere, and surely, no intelligent dentist need be told what they are. To what end does the reading, observation, and experience of the practicing dentist tend, if not to convince him of the fact that although dentistry has advanced wonderfully, yet it is hardly out of the domain of barbarism. Occasionally glimpses of the fair land of civilization may be caught through the forest, but a vigorous laying of the ax to the root of the tree is still needed, before the hardiest pioneer can hope to sit down among the dwellers in the palaces of science, and receive the homage of a grateful and admiring world. B.

